# Instructive Role of the Microenvironment in Preventing Renal Fibrosis

**DOI:** 10.5966/sctm.2016-0095

**Published:** 2016-10-05

**Authors:** Kei Matsumoto, Sandhya Xavier, Jun Chen, Yujiro Kida, Mark Lipphardt, Reina Ikeda, Annie Gevertz, Mario Caviris, Antonis K. Hatzopoulos, Ivo Kalajzic, James Dutton, Brian B. Ratliff, Hong Zhao, Zbygniew Darzynkiewicz, Stefan Rose‐John, Michael S. Goligorsky

**Affiliations:** ^1^ Department of Medicine, New York Medical College, Valhalla, New York, USA; ^2^ Department of Pharmacology, New York Medical College, Valhalla, New York, USA; ^3^ Department of Physiology, New York Medical College, Valhalla, New York, USA; ^4^ Department of Pathology, New York Medical College, Valhalla, New York, USA; ^5^ Renal Research Institute, New York Medical College, Valhalla, New York, USA; ^6^ Showa University, Tokyo, Japan; ^7^ Okayama University, Okayama, Japan; ^8^ Vanderbilt University, Nashville, Tennessee, USA; ^9^ University of Connecticut Health Center, Farmington, Connecticut, USA; ^10^ Stem Cell Institute, University of Minnesota, Minneapolis, Minnesota, USA; ^11^ Institute of Biochemistry, Christian‐Albrechts University, Kiel, Germany

**Keywords:** Transforming growth factor‐β1, α‐Smooth muscle actin, Green fluorescent protein, Leukemia inhibitory factor, Hyper‐interleukin‐6

## Abstract

Accumulation of myofibroblasts is a hallmark of renal fibrosis. A significant proportion of myofibroblasts has been reported to originate via endothelial‐mesenchymal transition. We initially hypothesized that exposing myofibroblasts to the extract of endothelial progenitor cells (EPCs) could reverse this transition. Indeed, in vitro treatment of transforming growth factor‐β1 (TGF‐β1)‐activated fibroblasts with EPC extract prevented expression of α‐smooth muscle actin (α‐SMA); however, it did not enhance expression of endothelial markers. In two distinct models of renal fibrosis—unilateral ureteral obstruction and chronic phase of folic acid‐induced nephropathy—subcapsular injection of EPC extract to the kidney prevented and reversed accumulation of α‐SMA‐positive myofibroblasts and reduced fibrosis. Screening the composition of EPC extract for cytokines revealed that it is enriched in leukemia inhibitory factor (LIF) and vascular endothelial growth factor. Only LIF was capable of reducing fibroblast‐to‐myofibroblast transition of TGF‐β1‐activated fibroblasts. In vivo subcapsular administration of LIF reduced the number of myofibroblasts and improved the density of peritubular capillaries; however, it did not reduce the degree of fibrosis. A receptor‐independent ligand for the gp130/STAT3 pathway, hyper‐interleukin‐6 (hyper‐IL‐6), not only induced a robust downstream increase in pluripotency factors Nanog and c‐Myc but also exhibited a powerful antifibrotic effect. In conclusion, EPC extract prevented and reversed fibroblast‐to‐myofibroblast transition and renal fibrosis. The component of EPC extract, LIF, was capable of preventing development of the contractile phenotype of activated fibroblasts but did not eliminate TGF‐β1‐induced collagen synthesis in cultured fibroblasts and models of renal fibrosis, whereas a receptor‐independent gp130/STAT3 agonist, hyper‐IL‐6, prevented fibrosis. In summary, these studies, through the evolution from EPC extract to LIF and then to hyper‐IL‐6, demonstrate the instructive role of microenvironmental cues and may provide in the future a facile strategy to prevent and reverse renal fibrosis. Stem Cells Translational Medicine *2017;6:992–1005*

**Significance Statement**

This study continued exploration of the beneficial effects of endothelial progenitor cells (EPCs) and their secretory products. Remarkable prevention of renal fibrosis was documented after treatment with EPC extract in two different fibrogenic models of chronic kidney disease. One of the products of EPC is leukemia inhibitory factor, which itself activated the STAT3 pathway and mitigated formation of myofibroblasts, whereas a more potent gp130/STAT3 agonist, a fusion protein hyper‐IL‐6, also prevented renal fibrosis. This line of investigations represents an early attempt to complement progenitor cell transplantation with treatments based on the administration of secretory products released by these cells.

## Introduction

Transcription factor‐mediated reprogramming of fibroblasts to mature specialized cells has captured significant attention since the description of induced pluripotency by Takahashi and Yamanaka [1]. Successive studies, each using the set of defined transcription factors, produced data on reprogramming of fibroblasts to neuronal cells, cardiomyocytes, or hematopoietic stem cells [2, 3, 4]. Reprogramming has also been shown in vivo by the local delivery of transcription factors for therapeutic purposes [5].

In parallel with these spectacular developments, an intriguing technology for cell reprogramming has emerged by exposing reversibly permeabilized cultured somatic cells to extracts obtained from lineage‐dissimilar cells. This approach has produced somatic cells reprogrammed with the extracts from mammalian oocytes, human T‐lymphocytes, or neuronal precursors [6, 7, 8, 9, 10].

Tissue fibrosis, the common final outcome of diverse chronic diseases, is a result of conversion of multiple resident cells, such as fibroblasts and fibrocytes, pericytes, and epithelial, or endothelial cells, into myofibroblasts [11, 12, 13, 14, 15]. The accumulation of myofibroblasts leads to excessive matrix deposition and fibrotic distortion of tissue architecture, eventuating in the loss of organ function. Given that a sizable proportion of fibrogenic myofibroblasts has been reported to be generated by the process of endothelial‐to‐mesenchymal transition [15], we initially inquired whether this process could be reversed. The first experimental evidence that this is possible has recently been presented in the case of myocardial ischemia [16].

Toward this end, we attempted to reprogram myofibroblasts to endothelial cells using cell‐free extract obtained from murine endothelial progenitor cells, both in vitro and in vivo. We used models of renal fibrosis, unilateral ureteral obstruction, and chronic phase of folic acid‐induced nephropathy to monitor the extent of fibrosis after injection of cell extract. The described procedures resulted in an approximately 50% reduction of fibrosis by preventing myofibroblast formation rather than conversion of myofibroblasts to endothelial cells. Leukemia inhibitory factor (LIF) was found to represent a component of the extract partially responsible for prevention and reversal of myofibroblast formation. A receptor‐independent ligand activating gp130/STAT3 signaling, a fusion protein hyper‐interleukin‐6 (hyper‐IL‐6), was able to prevent fibrosis in a process that temporarily induced pluripotency transcription factors.

## Materials and Methods

### Experimental Animals

All animal studies were performed in accordance with the National Institutes of Health (NIH) NIH Guide for the Care and Use of Laboratory Animals and were approved by the Institutional Animal Care and Use Committee at New York Medical College. α‐Smooth muscle actin (SMA)‐green fluorescent protein (GFP) transgenic mice on a C57BL/6J background were obtained from Dr. Ivo Kalajzic (University of Connecticut Health Center, Farmington, CT). Mice were originally developed by Dr. Jen‐Yue Tsai (National Eye Institute/NIH) and carry a regulatory sequence of *αSMA* gene spanning 1074 base pair (bp) of the 5′‐flanking region, the transcription start site, 48 bp of exon 1, the 2.5‐kbp intron 1, and the 15‐bp exon 2 of mouse α‐SMA. GFP is specifically expressed in both vascular and nonvascular smooth muscle cells [17, 18]. Studies were performed in 20‐week‐old αSMA‐GFP transgenic mice or wild‐type siblings. Mice were housed in the animal care facility of New York Medical College (25°C, 50% humidity, 12‐hour dark/light cycle) and had free access to food and water.

### Disease Models

The unilateral ureteric obstruction (UUO) procedure was performed according to the standard protocol [19]. Mice were anesthetized with isoflurane and placed in the lateral position under body temperature control. Surgery was performed through a left flank incision. The ureter was identified and tied at the level of the lower pole of the kidney with two separate silk ties.

Mice were injected with EPC extract diluted 1:2 (10 μl) or buffer subcapsularly into the lower pole of UUO kidney on day 0. Mice were euthanized on day 14. At the end of the study, the mice were anesthetized, blood was collected by cardiac puncture, and kidneys were excised. Renal tissue was divided and portions were snap‐frozen in liquid nitrogen or fixed in 4% paraformaldehyde/phosphate‐buffered saline (PBS).

To produce folic acid‐induced nephropathy (FAN), mice were intraperitoneally injected with folic acid (250 mg/kg) in 0.3 M NaHCO_3_. EPC extract (10 μl) was injected subcapsularly into the left kidney and buffer (10 μl) was injected into the right kidney on day 56 after induction of FAN. Mice were euthanized on day 84 after induction of FAN. At the end of the study, mice were anesthetized, blood was collected by cardiac puncture, and kidneys were excised. Renal tissue was divided and portions were snap‐frozen in liquid nitrogen or fixed in 4% paraformaldehyde/PBS.

### Isolation and Culture of Primary Renal Fibroblasts

Fibroblasts were isolated from kidneys dissected from 8‐week‐old α‐SMA‐GFP mice, according to the previously published protocol [20]. Briefly, cells were seeded onto a 1% gelatin‐coated Petri dish, covered with 4 ml of Dulbecco’s modified Eagle’s medium (DMEM; American Type Culture Collection, Manassas, VA, https://www.atcc.org) containing 20% fetal bovine serum (FBS), and incubated at 37°C in a humidified 5% CO_2_ atmosphere. The primary cultures used in this study were between passage (P) 2 and P3. Cells were treated with EPC extract, recombinant mouse LIF (100 pg/ml–10 ng/ml) (Thermo Fisher Scientific Life Sciences, Waltham, MA, http://www.thermofisher.com), vascular endothelial growth factor (VEGF) (1 ng/ml), transforming growth factor (TGF)‐β1 receptor blocker (SB431542) (5 μM) (Tocris Bioscience, Bristol, UK, https://www.tocris.com), recombinant human TGF‐β1 (5 ng/ml) (R&D Systems, Minneapolis, MN, http://www.rndsystems.com), or recombinant human TGF‐β1 (5 ng/ml) in the presence of EPC extract or LIF for 4 days.

Alternatively, to isolate primary renal fibroblasts, single‐cell suspension from mouse kidney was prepared by using enzymatic digestion with a mixture of collagenase H and collagenase/Dispase (Roche, Basel, Switzerland, http://www.roche.com). Endothelial cells were then depleted with magnetic Dynabeads (Thermo Fisher) coated with rat anti‐mouse CD31 antibody (BD Pharmingen, San Jose, CA, http://www.bdbiosciences.com). The remaining cells were collected, resuspended in DMEM (supplemented with 10% FBS), plated on 1% gelatin‐coated 100‐mm dishes, and cultured at 37°C in a CO_2_ incubator. Upon confluence, cells were subcultured in gelatin‐coated dishes at a 1:3 ratio and designated as passage 1 (P1). For experiments, the cells at P2 were used. The fibroblast origin of cultured cells at P2 was confirmed with antibodies for platelet‐derived growth factor receptor‐ β, α−SMA, F4/80, and CD31. NIH3T3 fibroblasts (American Type Culture Collection) were cultured in DMEM (American Type Culture Collection) with 10% FBS. Cells were treated with recombinant human TGF‐β1 (5 ng/ml) (R&D Systems) for 5 days to induce myofibroblastic phenotype. Primary renal fibroblasts and NIH3T3 fibroblasts were treated with recombinant human TGF‐β1 (5 ng/ml) alone or TGF‐β1 with EPC extract, LIF, or hyper‐IL‐6 for 6 hours, 24 hours, and 48 hours for quantitative polymerase chain reaction analysis.

Additional studies were performed by using Oct4‐GFP‐reporter fibroblasts obtained from transgenic mice expressing tamoxifen‐inducible Cre recombinase MerCreMer under the control of the endogenous Oct4 locus [21]. Oct4‐GFP fibroblasts were cultured in DMEM containing 1× penicillin/streptomycin, 1% nonessential amino acids, and 10% FBS at 37°C in 5% CO_2_; 1 µM 4 hydroxytamoxifen was added to the medium. The transgenic mice contain the conditional MerCreMer cassette inserted in the 3′ ‐untranslated region of the Oct4 locus and combined with the dual membrane‐bound fluorescent protein mTmG reporter. Cre recombination occurs only in cells that are actively expressing Oct4 and when tamoxifen is present. In these cells, there is an irreversible switch from expressing membrane‐bound dTomato to expressing membrane‐bound enhanced GFP.

Embryonic mouse EPCs isolated from embryos at E7.5–E7.8 of development were kindly provided by Dr. A. Hatzopoulos [22]. EPCs were plated on 0.1% gelatin‐coated plates and maintained at 37°C and 5% CO_2_ in DMEM culture medium containing 20% heat‐inactivated serum (55°C for 30 minutes; Thermo Fisher), 0.1 mmol/liter 2‐mercaptoethanol, 1 mmol/liter modified Eagle’s medium nonessential amino acids (Thermo Fisher), 100 U/ml penicillin and 100 μg/ml streptomycin, 2 mmol/liter l‐glutamine (Thermo Fisher), and 2 mmol/liter HEPES, pH 7.5.

### Preparation of EPC Extract

EPCs were trypsinized, washed in PBS and in cold lysis buffer (10 mM HEPES, pH 8.2, 50 mM NaCl, 5 mM MgCl_2_, 1 mM dithiothreitol, and protease inhibitor cocktail), sedimented at 800*g*, and resuspended in 1 volume of cell lysis buffer. Cells were sonicated on ice in 250‐μl aliquots until cells and nuclei were completely lysed, as judged by microscopical examination. The lysate was sedimented at 15,000*g* for 10 minutes at 4°C and the supernatant was divided into aliquots, frozen in liquid nitrogen, and stored for up to 6 months at −80°C [23].

### Histologic and Immunofluorescence Studies

Tissues fixed in 4% paraformaldehyde/PBS were embedded in paraffin and 4‐μm‐thick sections were stained with Masson’s trichrome. The extent of Masson’s trichrome‐positive area was digitally quantified by using Photoshop (Adobe Systems, San Jose, CA, http://www.adobe.com) and expressed as a percentage of the total area. All histologic analyses were performed by two investigators without knowledge of the origin of the slides, and the mean values were calculated.

For immunofluorescence studies, kidneys were fixed in 4% paraformaldehyde overnight, transferred to PBS containing 30% sucrose overnight, embedded in optimal cutting temperature medium (Tissue‐Tek; Sakura Finetek U.S.A., Inc., Torrance, CA, http://www.sakura‐americas.com/) and cryosectioned (10‐μm‐thick sections). Sections were permeabilized with 0.3% Triton X‐100 for 5 minutes, blocked with PBS‐bovine serum albumin (BSA) (1%) for 1 hour, and stained with primary antibodies at 4°C overnight. The primary antibodies were rat monoclonal antibody to CD31 (platelet endothelial cell adhesion molecule 1) (BD Pharmingen), rabbit polyclonal antibody against α‐SMA (Abcam, Cambridge, MA, http://www.abcam.com), and rat monoclonal antibody to F4/80 (eBioscience, San Diego, CA, http://www.ebioscience.com). We used AlexaFluor 594 conjugated goat anti‐rat (Thermo Fisher) secondary antibodies, according to the manufacturer’s recommendations. Nuclei were counterstained with Hoechst 33342. Images were obtained by using a compound Nikon microscope (TE‐2000U; Nikon, Tokyo, Japan, http://www.nikon.com/) equipped with a SPOT digital camera (SPOT Imaging, Sterling Heights, MI, http://www.spotimaging.com). At least five visual fields per specimen were analyzed for localization of endothelial and myofibroblast markers. CD31‐positive peritubular capillary density and the number of α‐SMA‐positive cells were quantified by using a grid method (266 squares) applied to images acquired at ×40 magnification.

### Cytokine and Chemokine Analysis of EPC Extract

The concentration of a panel of 15 cytokines and chemokines in EPC extract was measured with a Luminex IS100 analyzer and results analyzed by using appropriate curve‐fitting software (Luminex 100IS software version 2; Luminex Corp., Austin, TX, https://www.luminexcorp.com/). The concentration of selected cytokines and chemokines was measured by using a Milliplex map kit (EMD Millipore Corp., Billerica, MA, http://www.emdmillipore.com). The EPC extract or lysis buffer alone was used for cytokine/chemokine analysis. Measurements were performed by using premixed antibody beads (Milliplex), according to manufacturer’s instructions, and results were read by using Luminex‐200 against a standard curve. Quality controls were included in each assay.

### Streptolysin O Permeabilization and Cell Extract Treatment

Procedures followed our previously reported protocol [24]. Preliminary experiments showed that the optimal concentration of streptolysin O (SLO) (Sigma‐Aldrich, St. Louis, MO, http://www.sigmaaldrich.com) for NIH 3T3 cells using a concentration of 400 ng/ml resulted in permeabilization efficiency of >80%, as assessed by monitoring the uptake of a Mr 70,000 Texas red‐conjugated dextran (50 μg/ml; Thermo Fisher). Trypan blue exclusion assay showed that cell viability at this SLO concentration was minimally affected. Briefly, NIH 3T3 cells were washed twice in cold PBS and once in ice‐cold Ca^2+^‐ and Mg^2+^‐free Hank’s balanced salt solution (Thermo Fisher) and divided into aliquots, and SLO was added. Permeabilized cells were suspended in 100 μl of EPC extract containing an ATP‐regenerating system (1 mmol/liter ATP, 10 mmol/liter creatine phosphate, and 25 μg/ml creatine kinase; 100 μmol/L GTP; all from Sigma‐Aldrich), and 1 mmol/liter of nucleotide trisphosphate (Roche Diagnostics) and incubated for 1 hour at 37°C in a dry heat block with occasional agitation. To reseal cell membranes, the cell suspension was diluted with complete endothelial cell medium containing 2 mM CaCl_2_, and cells were cultured in endothelial basal medium‐2 (EBM‐2) supplemented with growth supplements (Lonza, South Plainfield, NJ, http://www.lonza.com). As a control, NIH 3T3 cells were cultured in EBM‐2 supplemented with growth supplements.

### Imaging of α‐SMA‐GFP cells

Primary renal fibroblasts obtained from wild‐type or transgenic mice were cultured on gelatin‐coated eight‐well culture slides (Lab‐Tek Chamber Slides, Thermo Fisher) and fixed with 4% paraformaldehyde for 10 minutes, permeabilized with 0.3% Triton X‐100 for 5 minutes, and blocked with 1% BSA/PBS for 1 hour. Cells were stained with anti‐CD144 (VE‐Cadherin) (BD Pharmingen), followed by incubation with secondary antibodies. Time‐lapse images were acquired by using IncuCyte Zoom (Essen BioScience, Ann Arbor, MI, http://www.essenbioscience.com) every 3 hours for 4 days without media change. Alternatively, imaging was performed by using an inverted fluorescence microscope (Nikon) with images captured using a Sony XC‐77 camera (Sony, Tokyo, Japan, http://www.sony.com). In addition, we used laser‐scanning cytometry to interrogate large numbers of cells [25].

### Quantitative PCR Analysis

Total RNA was isolated by using SpinSmart RNA Mini Purification Kit (Denville Scientific Inc., Metuchen, NJ, https://www.denvillescientific.com). One microgram of RNA was reverse transcribed by using a High Capacity RNA‐cDNA kit (Thermo Fisher). Real‐time quantitative PCR was performed by using Perfecta SYBR Green FastMix on a Stratagene MX3000P (Thermo Fisher).

The following primers were used: COL1‐FOR primer 5′CTG CTG GCA AAG ATG GAG A3′ and COL1‐REV primer 5′ACC AGG AAG ACC CTG GAA TC3′; COL3‐FOR primer 5′ CAA ATG GCA TCC CAG GAG3′ and COL3‐REV primer 5′CAT CTC GGC CAG GTT CTC 3′; α‐SMA‐FOR primer 5′AGC GTG AGA TTG TCC GTG ACA T3′ and α‐SMA‐REV primer 5′ GCG TTC GTT TCC AAT GGT GA3′; VE‐cadherin‐FOR primer 5′ACC ATC GCC AAA AGA GAG AC3′ and VE‐cadherin‐REV primer 5′TCT TGC CAG CAA ACT CTC CT3′; CD31‐FOR primer 5′AGC GCA GTC TTA CCG AAG G3′; CD31‐REV primer 5′TCT TGC CAG CAA ACT CTC CT3′; Oct4–FOR primer CAAGGCAAGGGAGGTAGACA; REV primer GCTCCTGATCAACAGCATCA; KLF4 – FOR primer CCAGCAAGTCAGCTTGTGAA; REV primer GGGCATGTTCAAGTTGGATT; c‐Myc – FOR primer GCCTAACCTCACAACCTTGG; REV primer CCTATTTACATGGGAAAATTGGA; Nanog – FOR primer AGCCTCCAGCAGATGCAAGA; and REV primer GCACTTCATCCTTTGGTTTTGA. Values were normalized for the abundance of the amplified 18s rRNA in each experiment. Fold change in gene expression was determined by using the 2‐ΔΔCT method.

### Statistical Analysis

All experiments were repeated at least three times. Data were analyzed using NCSS9 (NCSS Statistical Software, Kaysville, Utah; http://www.ncss.com) and are expressed as mean ± SEM. The data were examined by using analysis of variance with the Bonferroni post hoc test, Mann‐Whitney test, or Kruskal‐Wallis test. *p* < .05 was considered to represent statistically significant differences.

## Results

### Effects of EPC Extract on Fibroblast‐to‐Myofibroblast Transition

We cultured 3T3 fibroblasts in fibroblast medium and EBM2 endothelial medium; cells were permeabilized and treated with EPC extract. Only the combination of endothelial‐specific medium and EPC extract was sufficient to change the morphologic features of cultured fibroblasts into cells self‐organizing into a capillary‐like web (supplemental online Fig. 1) within 28 days of such a treatment. In view of the prior evidence of fibroblast reprogramming into endothelial cells [26], we next examined the expression of endothelial markers (VE‐cadherin) in cultured fibroblasts exposed to EPC extract. Despite the ability of these cells to form capillary‐like tubes, the expression of endothelial markers was marginal, thus calling into question their reprogramming toward endothelial lineage. Immunohistochemical characterization of the treated cells, however, showed a significant reduction in α‐SMA staining (supplemental online Fig. 2). In addition, we attempted to reproduce effects of the EPC extract by using similarly prepared extract of mature endothelial cells. This procedure failed to appreciably change the phenotype of fibroblasts and reproduce the results obtained with the EPC extract (data not shown).

**Figure 1:**
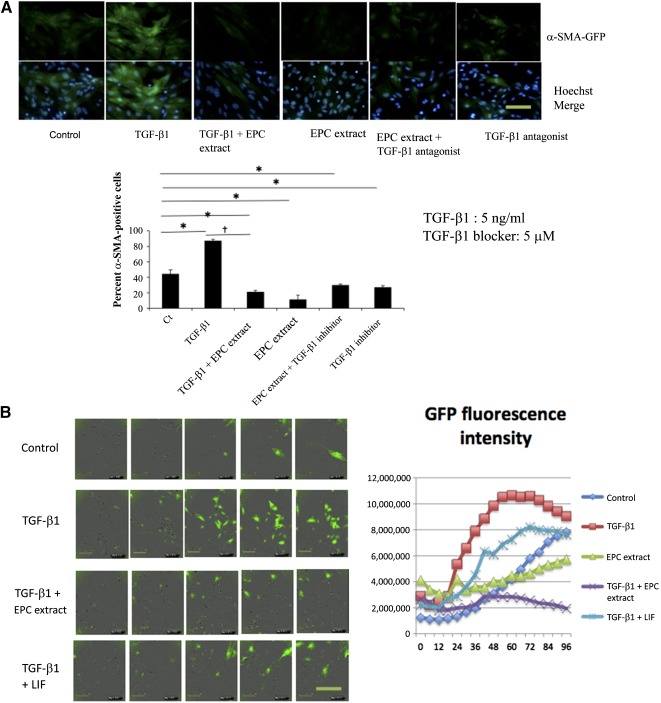
Immunocytochemical detection of α‐SMA in National Institutes of Health 3T3 cells (*n* = 3) **(A)** and time‐lapse videomicroscopy images of α‐SMA‐GFP‐positive fibroblasts **(B)** after activation with TGF‐β1 in the presence of EPC extract or LIF (vide infra). ∗, *p* < .05 versus control; †, *p* < .05 versus TGF‐β1. Original magnification, ×40. Scale bars = 25 μm. Data on time‐lapse videomicroscopy is representative of two independent experiments. Abbreviations: α‐SMA, α‐smooth muscle actin; EPC, endothelial progenitor cell; GFP, green fluorescent protein; LIF, leukemia inhibitory factor; TGF‐β1, transforming growth factor‐β1.

When fibroblasts were derived from α‐SMA‐GFP mouse kidneys (or NIH 3T3 fibroblasts used interchangeably), cells initially showed marginal expression of GFP (Fig. 1A), but the proportion of GFP‐positive cells increased dramatically after TGF‐β1 treatment. Under these conditions, when cells also received EPC extract through use of the permeabilization protocol, their conversion to α‐SMA‐positive myofibroblasts was significantly halted. We performed 96‐hour time‐lapse videomicroscopy of fibroblasts obtained from α‐SMA‐GFP mouse kidneys and omitted prior cell permeabilization. Under control conditions, approximately 20% of cells exhibited expression of GFP fluorescence. In contrast, nearly 80% of TGF‐β‐treated fibroblasts acquired GFP fluorescence within 24–48 hours (Fig. 1B). Cotreatment with the EPC extract resulted in approximately 33% of fibroblasts gaining GFP fluorescence (i.e., halting fibroblast‐to‐myofibroblast transition by nearly 50%). Without TGF‐β1 pretreatment, effects of the EPC extract alone were minimal or undetectable, suggesting that the TGF‐β1‐induced activation of fibroblasts could be abrogated by the EPC extract. Charted fluorescence intensity is summarized in Figure 1B. Collectively, these findings suggested modulation of fibroblast cell fate by the EPC extract.

### In Vivo Effects of EPC Extract in Models of Renal Fibrosis

Unexpectedly, the EPC extract‐mediated inhibition of TGF‐β1‐induced phenotypic changes did not require prior cell permeabilization, as shown in data presented in Figure 1B. This raised the question: Is the effect of EPC extract mediated via (a) changes in the extracellular environment, (b) receptor‐mediated signaling by the components of the extract, or (c) temporary spontaneous permeabilization of the plasma membrane in the course of transition from a fibroblast to a myofibroblast? In fact, mechanically active cells undergo transient permeabilization [27, 28], thus buttressing the third explanation while not rejecting the former ones (see following discussion). Collectively, these studies have established the possibility of preventing in vitro TGF‐β1‐induced fibroblast activation and acquisition of the myofibroblastic phenotype using EPC extract treatment of nonpermeabilized cells and raised the possibility of transcendence of this effect to an in vivo setting.

In the light of potential therapeutic significance of the preceding in vitro observation that EPC extract‐mediated prevention of fibroblast‐to‐myofibroblast transition can be achieved without prior cell permeabilization, we next attempted to test this effect in vivo. We argued that cell permeabilization could be omitted because even under physiologic conditions diverse cells undergo this process, especially in a mechanically active microenvironment [27, 28]. Indeed, when 5 days after UUO kidneys were injected subcapsularly with 70 kD Texas red dextran, the agent was readily detectable in the renal parenchyma of the UUO but not the contralateral kidney 1 hour after the injection (supplemental online Fig. 3).

**Figure 2:**
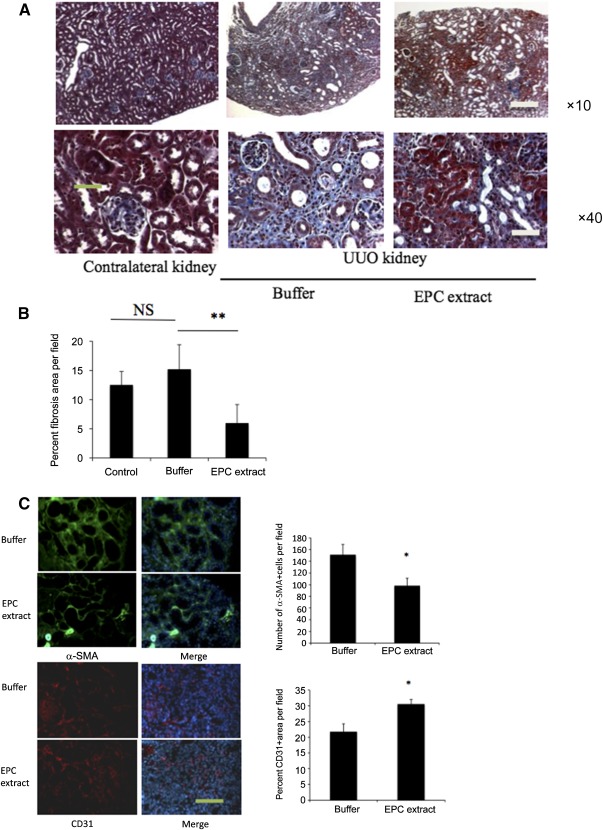
Endothelial progenitor cell extract reduces fibrosis in UUO kidney. EPC extract was injected subcapsularly, as detailed in Materials and Methods. **(A):** Renal histology on day 14 in vehicle‐treated and EPC extract‐treated kidneys. Original magnification, ×10, ×40. **(B):** Quantitative assessment of interstitial fibrosis in each group. Each group contained seven mice, and five fields per mice were evaluated in a blinded to the source manner. Data are expressed as mean ± SEM (Kruskal‐Wallis test). ∗∗, *p* < .01 versus UUO mice with the same duration of vehicle treatment. (C): Subcapsular injection of EPC extract to UUO kidneys reduced the number of α‐SMA‐GFP‐positive cells and improved the density of peritubular capillaries. ∗, *p* < .05. Scale bars = 400 and 100 μm **(A)** and 100 μm **(C)**. Abbreviations: α‐SMA, α‐smooth muscle actin; EPC, endothelial progenitor cell; NS, not significant; UUO, unilateral ureteric obstruction.

On the basis of these findings, we next performed UUO, the standard model of renal fibrosis, and injected the affected kidney with EPC extract or the vehicle. Subcapsular injection of the EPC extract into UUO kidneys was associated with the nearly 50% reduction of renal fibrosis (Fig. 2A). The number of myofibroblasts was reduced by 35% and CD31‐positive area was increased by 35% compared with those in vehicle‐treated mice (Fig. 2B). EPC extract treatment was also associated with the reduction of macrophage infiltration of UUO kidneys (supplemental online Fig. 4). Considering the breadth of EPC extract effects, it appears less likely that its observed actions were mediated via myofibroblast permeabilization.

**Figure 3:**
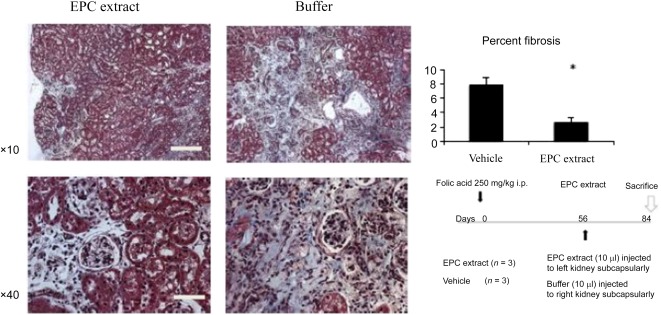
Subcapsular injection of EPC extract into kidneys with folic acid‐induced nephropathy resulted in a significant reversal of fibrosis. Masson’s trichrome staining of kidney sections, and analysis of blue pixels. *n* = 3. ∗, *p* < .05. Right lower panel shows the chronological relations between the initiation of disease (folic acid injection), subcapsular injection of the EPC extract, and the termination of the experiment. Abbreviation: EPC, endothelial progenitor cell.

In another model of renal fibrosis, the chronic phase of folic acid‐induced nephropathy, α‐SMA‐GFP transgenic mice were injected intraperitoneally with folic acid (250 mg/kg) on day 0. EPC extract (10 μl) was injected subcapsularly in left kidneys and the vehicle (10 μl) was injected in right kidneys on day 56 after the induction of folic acid nephropathy. Notably, at this time, post‐folic acid induction renal fibrosis becomes readily detectable [29]. Mice were sacrificed 3 months after initiation of folic acid‐induced nephropathy. As summarized in Figure 3, this delayed treatment with EPC extract resulted in a remarkable reversal of renal fibrosis. Hence, the in vivo data demonstrated that (a) the effect of EPC extract is not limited to the UUO model and (b) the EPC extract is capable of not only preventing but also reversing established tubulointerstitial fibrosis. Remarkably, the amelioration of renal fibrosis was associated with the restoration of microvascular density. It is doubtful, however, that this finding is due to the reversal of endothelial‐mesenchymal transition because we were unable to detect endothelial markers in cultured fibroblasts treated with the EPC extract. It is consistent, however, with the previous reports on the proangiogenic effect of fibroblasts [30, 31].

### Cytokine and Chemokine Screen of EPC Extract Revealed LIF as a Putative Effector

**Figure 4:**
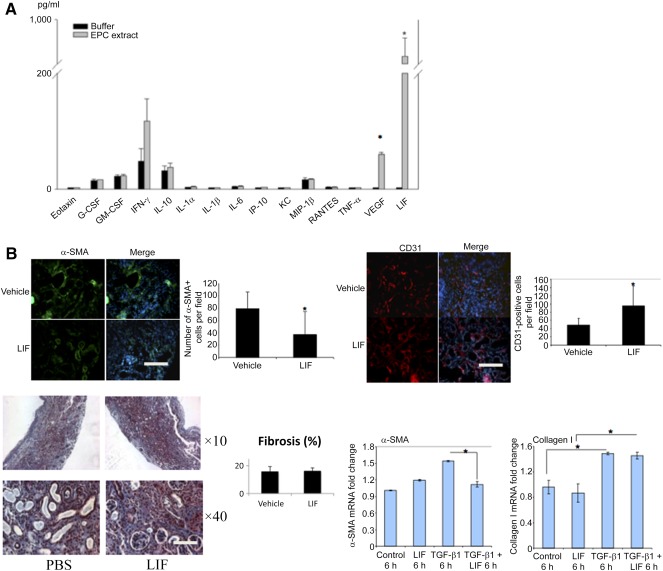
LIF abundance in the EPC extract and its actions in vivo. (**A):** Results of the multiplex analysis of the abundance of different cytokines and chemokines in the EPC extract (triplicate samples). Results are compared with the nonconditioned medium. (**B):** The number of α‐smooth muscle actin (α‐SMA)‐green fluorescent protein‐positive cells is reduced (top left) and the density of peritubular capillaries is increased (top right), but fibrosis remains unchanged in kidneys of mice with UUO (lower left), which received subcapsular injection of LIF, compared with the vehicle alone (*n* = 4–6 mice per group). In lower right, quantitative reverse‐transcriptase polymerase chain reaction data on the abundance of α‐SMA and collagen I are consistent with morphologic findings. ∗, *p* < .05. Scale bars = 100 μm. Abbreviations: EPC, endothelial progenitor cell; G‐CSF, granulocyte colony‐stimulating factor; GM‐CSF, granulocyte‐macrophage colony‐stimulating factor; IFN, interferon; IL, interleukin; IP, inducible protein; KC, keratinocyte chemoattractant; LIF, leukemia inhibitory factor; MIP, macrophage inflammatory protein; PBS, phosphate‐buffered saline; RANTES, regulated on activation, normal T‐cell expressed and secreted; TNF, tumor necrosis factor; VEGF, vascular endothelial growth factor.

We next inquired what components of EPC extract could mediate the observed effects. Multiplex analysis of EPC extract showed that it was dramatically enriched in LIF and VEGF, much above other cytokines and chemokines (Fig. 4A). Notably, the elevation of LIF was unique to these cells, as our previous studies did not detect it in these amounts in the conditioned media of mesenchymal stem cells or macrophages [32]. Moreover, recent proteomic studies of endothelial cell secretome did not detect prodigious amounts of LIF (manuscript in preparation). These findings raised the possibility that LIF and VEGF could be responsible for the observed actions of EPC extract. Of note, the previous study of the transcriptome of these EPC revealed upregulation of these messages [33]. When we substituted EPC extract in the cell treatment protocol with LIF or VEGF, only LIF (100 pg/ml, the concentration similar to that seen in the extract) was able to mimic the effect of the EPC extract in halting conversion to myofibroblasts (Figure 1B and data not shown). The effect of LIF in preventing formation of myofibroblasts was evident after 24 hours but dissipated at later times. These in vitro findings of effects of EPC extract components led to attempts to reproduce these results in vivo. Injection of LIF to UUO kidney (100 pg LIF/kidney in 10 μl of PBS), at the time of the surgery or 1 week after the surgery, resulted in the substantially reduced number of α‐SMA‐GFP cells by LIF injection to UUO kidneys (Fig. 4B), and microvascular rarefaction, a hallmark of UUO, was significantly prevented (Fig. 4C). In contrast to this, injection of LIF did not result in any appreciable decline in the degree of fibrosis commensurate with that seen upon injection of EPC extract. The similar lack of antifibrotic effect of LIF alone was detected in the chronic phase (3 months after induction) of the folic acid nephropathy model (supplemental online Fig. 5). These findings raise the possibility of dissociation between myofibroblastogenesis and the degree of fibrosis, at least within the timeframe of current experiments.

### Therapeutic Induction of gp130‐STAT3 Pathway Mitigated Fibrosis

**Figure 5:**
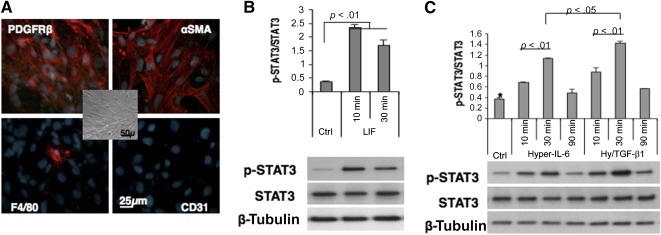
Effects of LIF and hyper‐IL‐6 on STAT3 phosphorylation in primary cultures of renal fibroblasts. **(A):** Immunocytochemical demonstration of the purity of renal fibroblast cultures devoid of CD31‐positive and containing only rare F4/80‐positive cells (a bright‐field image is presented in the center panel). **(B):** LIF‐induced phosphorylation of STAT3. **(C):** Hyper‐IL‐6‐induced phosphorylation of STAT3 in the presence or absence of TGF‐β1. Note that maximal effect is achieved when both hyper‐IL‐6 and TGF‐β1 are applied. LIF was used at the concentration of 100 pg/ml, hyper‐IL‐6 at 10 ng/ml; *n* = 3–4 per group. Abbreviations: αSMA; α‐smooth muscle actin; Ctrl, control; IL, interleukin; LIF, leukemia inhibitory factor; PDGFRβ, platelet‐derived growth factor receptor β.

**Figure 6:**
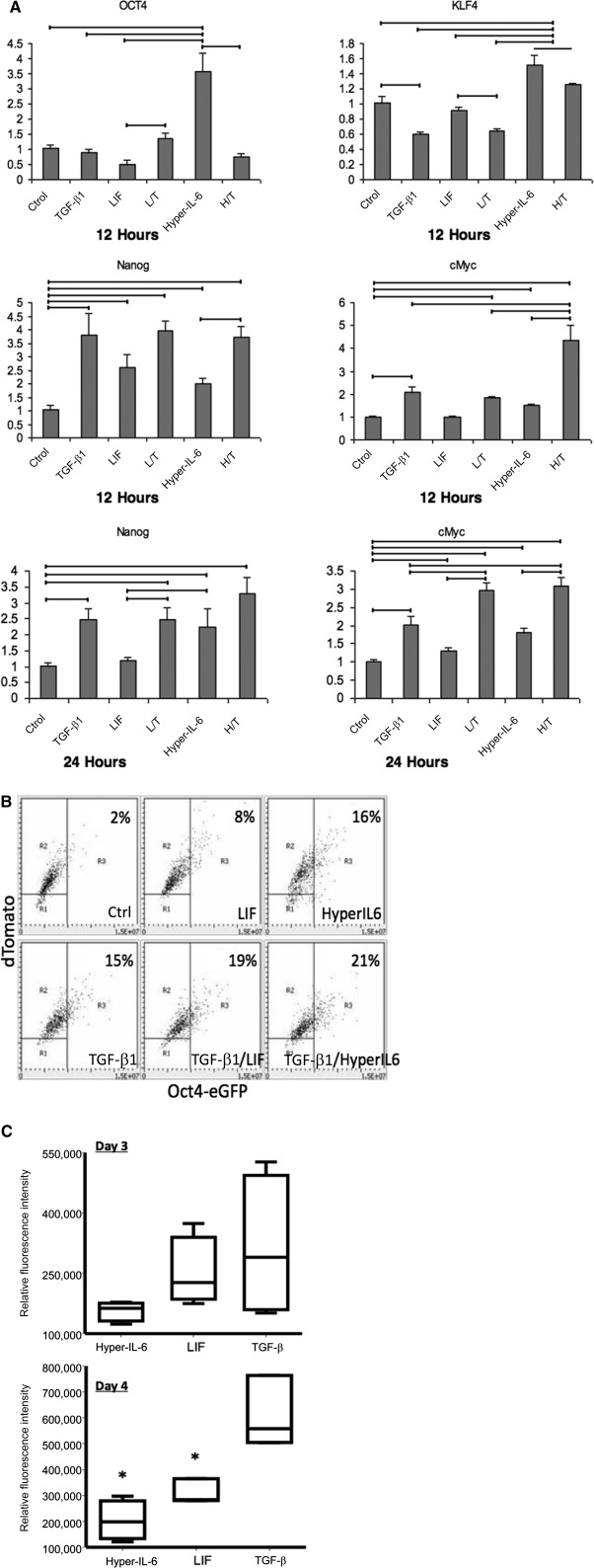
Analysis of pluripotency transcription factors Nanog and c‐Myc after application of LIF or hyper‐IL‐6 in the presence or absence of TGF‐β1 and myofibroblast‐to‐fibroblast transition induced by LIF and hyper‐IL‐6. **(A):** Results of quantitative polymerase chain reaction. After 24 hours, only c‐Myc and Nanog showed significant differences (Oct4 and Klf4 are not shown). Horizontal lines, statistically significant differences between the groups (*p* < .05). **(B):** Laser scanning cytometry [25] data obtained in two separate experiments, each in duplicate, using Oct4‐GFP reporter fibroblasts. **(C):** Box‐and‐whiskers plot of myofibroblast‐to‐fibroblast transition induced by LIF and hyper‐IL‐6. Primary renal fibroblasts isolated from α‐smooth muscle actin (α‐SMA)‐green fluorescent protein (GFP) mice were cultured on Matrigel‐coated plastic dishes for 96 hours. TGF‐β1 (5 ng/ml) induced myofibroblastic transition after 48 hours. By 96 hours, fluorescence intensity of α‐SMA‐GFP was significantly reduced in LIF‐ and hyper‐IL‐6‐treated groups (*n* = 4 each). ∗, *p* < .05, Kruskal‐Wallis test. Abbreviations: Ctrl, control; eGFP, enhanced green fluorescent protein; H/T, hyper‐interleukin‐6–transforming growth factor‐β1; hyper‐IL‐6; hyper‐interleukin‐6; LIF, leukemia inhibitory factor; L/T, leukemia inhibitory factor–transforming growth factor‐β1; TGF‐β1, transforming growth factor‐β1.

To unravel the apparent lack of antifibrogenic in vivo LIF effect, we recreated the experiment in vitro and examined the expression of known downstream targets of LIF/gp130 signaling via STAT3 (Klf‐4, c‐Myc, Oct4, and Nanog [34, 35, 36]). We used primary cultures of renal fibroblasts that showed a high degree of purity and no endothelial contamination (Fig. 5A). We first confirmed STAT3 activation in these cells. LIF resulted in a fast wave of STAT3 phosphorylation (Fig. 5B). A similar STAT3 phosphorylation was achieved by using a receptor‐independent gp130 ligand, a fusion protein hyper‐IL‐6 [37] (Fig. 5C), consisting of IL‐6 fused via a 40‐Å linker to the soluble extracellular portion of the IL‐6 receptor. Quantitative PCR analysis of transcription factors (Fig. 6) showed that at 12 and 24 hours after administration of LIF or hyper‐IL‐6, the abundance of message for Nanog and c‐Myc was elevated and coapplication of LIF or hyper‐IL‐6 with TGF‐β1 resulted in a further increase of mRNA encoding these transcription factors within the preceding time frame. In the case of c‐Myc, induction was significantly more pronounced at 12 hours after application of hyper‐IL‐6. These findings suggest the acquisition by myofibroblasts of transient pluripotency induced by gp130/STAT3 activation.

To resolve uncertainty related to changes in Oct4, we next performed experiments in Oct4‐GFP‐reporter fibroblasts obtained from transgenic mice expressing tamoxifen‐inducible Cre recombinase MerCreMer under the control of the endogenous Oct4 locus developed by Dutton’s group [21]. Under basal conditions with tamoxifen application only, GFP fluorescence was nearly undetectable in a large cohort of cells examined by using laser scanning cytometry (Fig. 6B). Both LIF and hyper‐IL‐6 resulted in a moderate induction of Oct4‐GFP, as did TGF‐β alone. However, only application of TGF‐β in combination with LIF and, to a higher degree, hyper‐IL‐6 produced 1/5‐1/4 conversion to Oct4‐expressing cells.

We next inquired whether treatment of myofibroblasts with LIF or hyper‐IL‐6 could reverse myofibroblasts to fibroblasts. To address this possibility, we cultured primary renal fibroblasts isolated from α‐SMA‐GFP mice on Matrigel‐coated plastic (Corning Inc., Corning, NY, http://www.corning.com) for 96 hours and induced myofibroblastic transition by adding TGF‐β1 (5 ng/ml) to cultured cells. After 48 hours, when the proportion of myofibroblasts increased significantly, as judged by the enhancement of α‐SMA‐GFP fluorescence. As summarized in Fig. 6C, at 72 hours there was no statistically significant difference in fluorescence intensity between cells subjected to TGF‐β1 alone or in combination with LIF or hyper‐IL‐6; however, at 96 hours, fluorescence intensity of α‐SMA‐GFP was significantly reduced in the treated groups. The data suggest the possibility of myofibroblast‐to‐fibroblast transition induced by LIF or hyper‐IL‐6.

**Figure 7:**
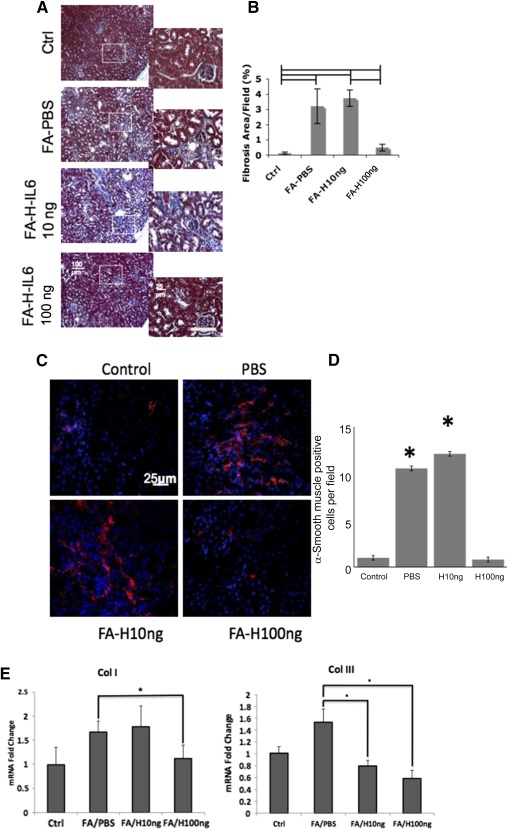
Intrarenal injection of hyper‐IL‐6 results in a dose‐dependent amelioration of renal fibrosis **(A, B)**, reduction in the number of α‐smooth muscle actin‐positive cells **(C, D)**, and mRNA encoding col I and III **(E)** in the FA‐induced nephropathy. Data are the mean ± SEM of *n* = 3–5. Lines, *p* < .05. Scale bar = 100 μm **(A)**. Abbreviations: Col, collagenase; Ctrl; control; FA, folic acid; H10ng, hyper‐IL6 10 ng; H100ng, hyper‐IL6 100 ng; PBS, phosphate‐buffered saline.

On the basis of these findings, we next examined the in vivo effect of hyper‐IL‐6 in the model of chronic folic acid‐induced nephropathy (the duration of this model allows for the delayed institution of treatments). As shown in Figure 7A, renal subcapsular injection of hyper‐IL‐6 1 week after induction of nephropathy resulted in a dramatic dose‐dependent prevention of fibrosis 3 months later (using 100 ng hyper‐IL‐6 per injected kidney, but not 10 ng). This effect was associated with a significant decline in the number of α‐SMA‐positive fibroblasts (Fig. 7C, 7D). Quantitative PCR analysis of the kidneys showed the reduction of collagen I and III transcripts (Fig. 7E).

## Discussion

The data presented herein show that cell‐free extract of endothelial progenitor cells, but not the extract of mature endothelial cells, reprograms kidney‐derived and 3T3 fibroblasts to prevent their TGF‐β1‐induced acquisition of myofibroblastic phenotype in culture. Moreover, extracts obtained from endothelial progenitors and injected into fibrotic kidneys ameliorate development of fibrosis. LIF and VEGF, both highly expressed in the extract of endothelial progenitors, were tested in in vitro reprogramming of myofibroblasts and only LIF was found to be effective. However, it was not sufficient to affect the course of fibrotic disease in vivo, probably indicating that additional reprogramming factors, either exogenous or endogenous, are necessary for antifibrogenic activity of the EPC extract. Nonetheless, LIF administration in vivo in two different models of nephrosclerosis curtails myofibroblastic transformation in a way similar to in vitro data. Induction of STAT3 pathway and pluripotency transcription factors were incriminated in the effect of LIF on primary fibroblasts. Another, receptor‐independent, ligand of gp130, hyper‐IL‐6, more robustly induces STAT3 and pluripotency transcription factors Oct4, KLF4, and c‐Myc. In vivo, renal subcapsular injection of hyper‐IL‐6 exhibits a profound antifibrotic effect. Thus, commencing with the cell extract and observing its antifibrotic action, studies progressed toward identification of an active component of the extract, LIF, which partially mimics the effect of the extract; the studies evolved to a more potent gp130 ligand, hyper‐IL‐6, to disclose its robust antifibrotic effect in the kidney.

The previously held views of cell‐autonomous mechanisms defining its fate and state of differentiation have recently been challenged, thus bringing to the fore the mechanisms of cell‐cell communication and the instructive role of the microenvironment [38, 39, 40]. Conversion of adoptively transferred mesenchymal stem cells into endothelial cells in the ischemic heart has been described [41]. Nanofibers preconditioned with embryonic stem cell secretome and injected into injured kidney have a renoprotective effect [42]. Hence, the long‐term objective of our studies is to define the active components of the EPC extract that could mimic its antifibrotic properties. The first in a series screen of the EPC extract detecting cytokines and chemokines reveals that it is highly enriched in LIF and VEGF.

Indeed, application of LIF alone to TGF‐β1‐stimulated cultured fibroblasts prevented and reversed their transition to myofibroblasts. In in vivo experiments using two distinct models of tubulointerstitial fibrosis, LIF again prevented accumulation of myofibroblasts; however, it failed to prevent development of fibrosis. LIF/gp130/STAT3 pathway is known to be sufficient to maintain an undifferentiated state of mouse embryonic stem cells [43]. LIF has been found to control dedifferentiation of embryonic epiblasts to inner cell mass‐like cells [44] and is routinely used in mouse reprogramming media [45]. Survival of cardiomyocytes and cardiac regeneration are enhanced by LIF [46]. Most recently, LIF has been shown to attenuate renal fibrosis [47], but daily injections are required. Our own data demonstrate that LIF not only is highly enriched in EPC extract but possesses anticontractile functions, without perturbing matrix synthesis in TGF‐β1‐stimulated fibroblasts. Admittedly, our data do not cover the broad range of possible doses and regimens of administration. This dichotomy is consistent with the separate regulatory mechanisms for α‐SMA and collagen 1 synthesis by TGF‐β1‐stimulated fibroblasts.

It is curious that the therapeutic prevention and reversal of fibroblast‐to‐myofibroblast transition do not require cell permeabilization. There is growing awareness, based on a series of studies from McNeil and Steinhardt [27, 28], of spontaneous bouts of permeabilization of the plasma membrane occurring in most cells, but mechanically active cells have a much higher probability of permeabilization. This is precisely the case with the contractile cells, such as myofibroblasts, which become readily loaded with the 70‐kD dextran, whereas interstitial fibroblasts (which are less active mechanically) are not. This fact alone makes myofibroblasts selectively amenable for therapeutic targeting without prior permeabilization and offers facile technical advantages over other reprogramming techniques, such as cell fusion and induction with transcription factors or small molecules, which lack cell‐type tropism. Whether this particular mechanism plays a critical role in the panoply of observed actions remains questionable because some of these effects are LIF‐receptor‐mediated whereas others that bypass the receptors and activate the gp130/STAT3 pathway, such as hyper‐IL‐6, do not require the permeabilization step.

Endothelial‐mesenchymal transition is a physiological developmental process of embryogenic formation of heart valves and septa, but some investigators argue that in adulthood this transition is a major contributor to vascular dropout and development of tubulointerstitial fibrosis [15, 48, 49]. From 30% to 50% of interstitial fibroblasts in fibrotic renal disease models originate from the endothelium. Although our original hypothesis has been based on the possibility of EPC extract‐induced transdifferentiation of myofibroblasts toward endothelial lineage, the data obtained are more consistent with the conversion of myofibroblasts into fibroblasts. In fact, such a myofibroblast‐to‐fibroblast conversion has been previously described [50], and it has been shown that amniotic membrane stromal extract can actuate this reversal [51]. Nevertheless, administration of the EPC extract was accompanied by improvement in microvascular density, but this effect does not appear to be secondary to mesenchymal‐endothelial transition; rather, it is most likely attributed to the fibroblast‐promoted angiogenesis, as previously demonstrated [30, 31].

The finding that LIF and, to a greater extent, hyper‐IL‐6 activate gp130/STAT3 pathway with the subsequent induction of pluripotency factors (c‐Myc, Nanog, and later Oct4) raises the possibility of the acquisition of a transitional stage during reversal of myofibroblasts to fibroblasts via a ground‐state of a pluripotent cell. It is not excluded that, given the proper differentiation cues, such cells may differentiate toward diverse lineages. In fact, more than 2 decades ago Kato and Gurdon [52] established that *Xenopus* egg cytoplasm has a capacity for reprogramming somatic cell nuclei. Although this line of evidence would support the idea of reprogramming of our myofibroblasts by EPC extract, it is not necessarily the only pathway for the observed effect of hyper‐IL‐6, which is believed to rely entirely on the gp130 signaling. It is noteworthy that, when this article was in preparation, a transient acquisition of pluripotency during transdifferentiation of somatic cells with a mixture of transcription factors was described as a hallmark of reprogramming [53, 54]. In the context of our study, the similar conclusion can be reached when reprogramming is conducted via activation of gp130/STAT3 pathway rather than by supplying the downstream transcription factors. It is also remarkable that two known antifibrogenic strategies, IL‐10 and interferon‐γ, both activate the gp130/STAT3 pathway to achieve this effect [55, 56]. Certainly, fate‐mapping studies will be required in the future to monitor the trajectory of myofibroblasts in fibrotic kidneys treated with hyper‐IL‐6. Nonetheless, the very finding of induction of the pluripotency transcription factors, albeit in vitro, argues strongly in favor of transient pluripotency as a potentially notable contributor to reprogramming.

## Conclusion

EPC extract prevents and reverses fibroblast‐to‐myofibroblast transition and renal fibrosis. The component of EPC extract, LIF, is capable of preventing development of the contractile phenotype of activated fibroblasts but does not eliminate TGF‐β1‐induced collagen synthesis in cultured fibroblasts and renal fibrosis. Unquestionably, this component of EPC extract is a participant in its antifibrotic effect, but not the sole one, as other yet‐undisclosed factors may contribute to the antifibrotic effect of the extract. These findings should stimulate future identification and in‐depth characterization of such components. On the other hand, a receptor‐independent inducer of gp130/STAT3 pathway, hyper‐IL‐6, is capable of consistently inducing in vitro pluripotency transcription factors, Nanog and c‐Myc, and later on Oct4, and preventing renal fibrosis in vivo.

## Author Contributions

K.M., S.X., and J.C.: experiment design and execution, data analysis, review and approval of final manuscript; Y.K. and M.L.: experiment execution, data analysis, review and approval of final manuscript; R.I., A.G., M.C., B.B.R., H.Z., and Z.D.: experiment execution, review and approval of final manuscript; A.K.H.: generation of cells used, review and approval of final manuscript; I.K. and J.D.: generation of mice used, review and approval of final manuscript; S.R.‐J.: hyper‐IL‐6 synthesis, review and approval of final manuscript; M.S.G.: experiment design, manuscript writing.

## Disclosure of Potential Conflicts of Interest

The authors indicated no potential conflicts of interest.

## Supporting information

Supporting Information
