# Supplementary material for: Instructive Role of the Microenvironment in Preventing Renal Fibrosis
Source: Stem Cells Transl Med. 2016 Oct 5;6(3):992–1005. doi: 10.5966/sctm.2016-0095 (PMC5442777; doi:10.5966/sctm.2016-0095)
Supplement: Supplementary file 1 — Supporting Information [file SCT3-6-0992-s001.pdf]

SUPPLEMENTARY FIGURES

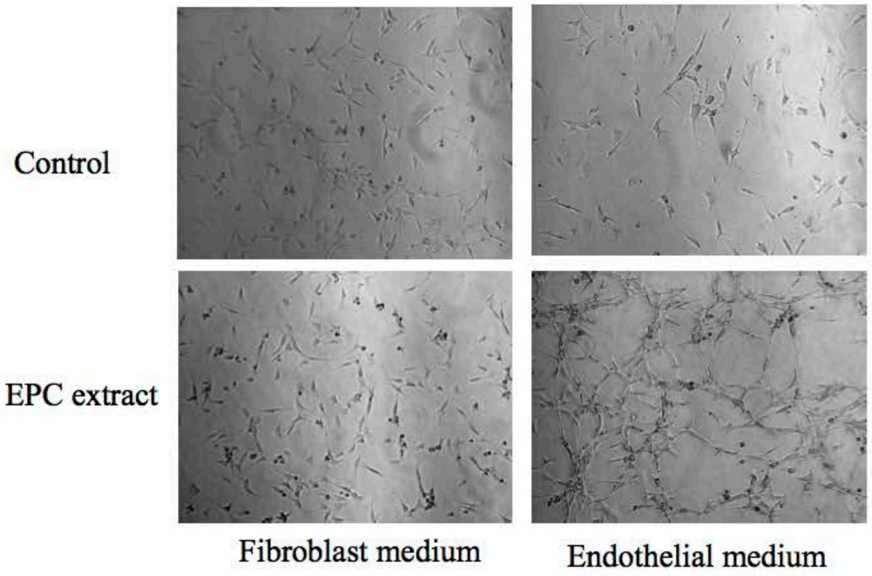

Suppl Fig.1. Bright-field images of cell morphology of NIH 3T3 cells treated with EPC extract and maintained in fibroblastic or endothelial-specific culture medium. Images were acquired on day 28 of observation. Note that only cells treated with EPC extract and maintained in endothelial medium formed capillary-like web structures.

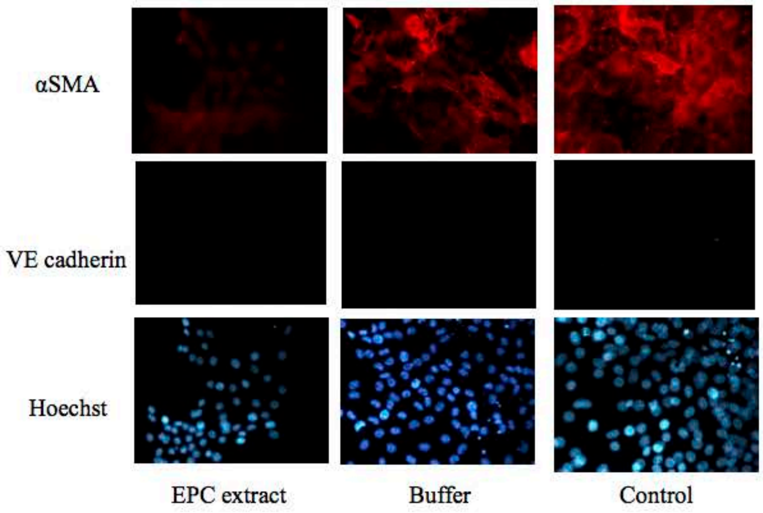

Suppl. Fig.2. Immunocytochemical detection of mesenchymal and endothelial markers in NIH 3T3 cells treated during transient permeabilization with the EPC extract, buffer alone or untreated. Note that on day 28 post-treatment only EPC extract-treated cells exhibited depletion of alpha-SMA. Neither group showed any appreciable appearance of endothelial marker VE-cadherin. Magnification x400.

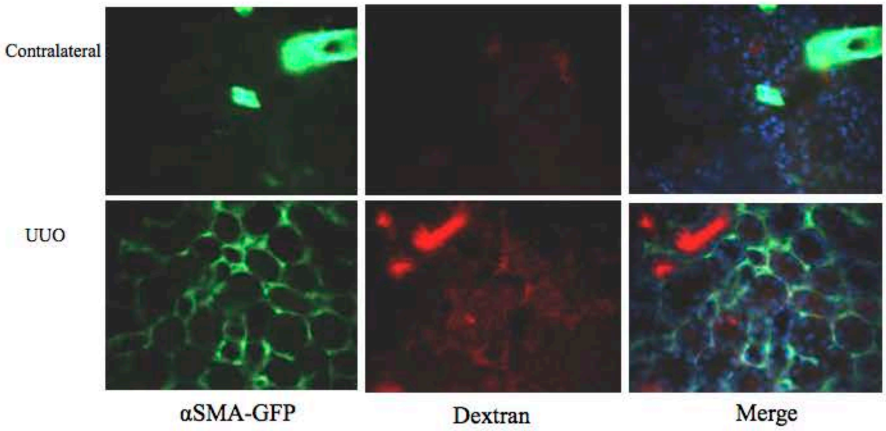

Suppl Fig 3. Detection of spontaneous permeabilization of myofibroblasts in UUO kidney, but not in the contralateral kidney, using Texas red-conjugated 70 kD dextran. Ten microliters of 500 ug/ml dextran was injected to both kidneys of alpha-SMA-GFP mice on day 5 post-UUO and animals euthanized 1 h later for detection of this macromolecular probe. Note the abundance of myofibroblasts (green) co-labeled with Texas red dextran (red) in UUO kidney, but not in the contralateral kidney.

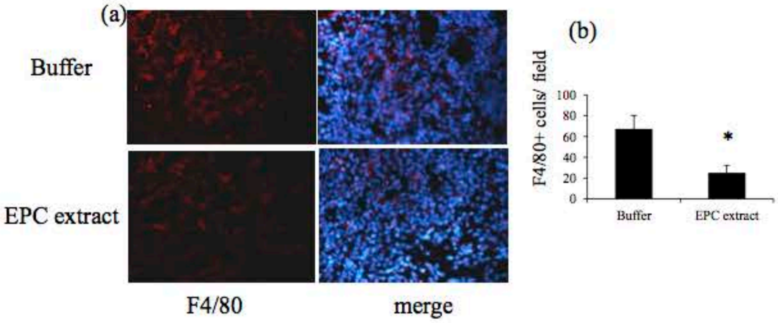

Suppl. Fig 4. Injection of EPC extract to UUO kidneys reduces infiltration by F4/80-positive macrophages. (a) Representative images. Nuclear staining is shown in merged images. (b) Quantitative summary. Asterisk denotes $p<0.05$ versus buffer-treated kidneys. Magnification x400.

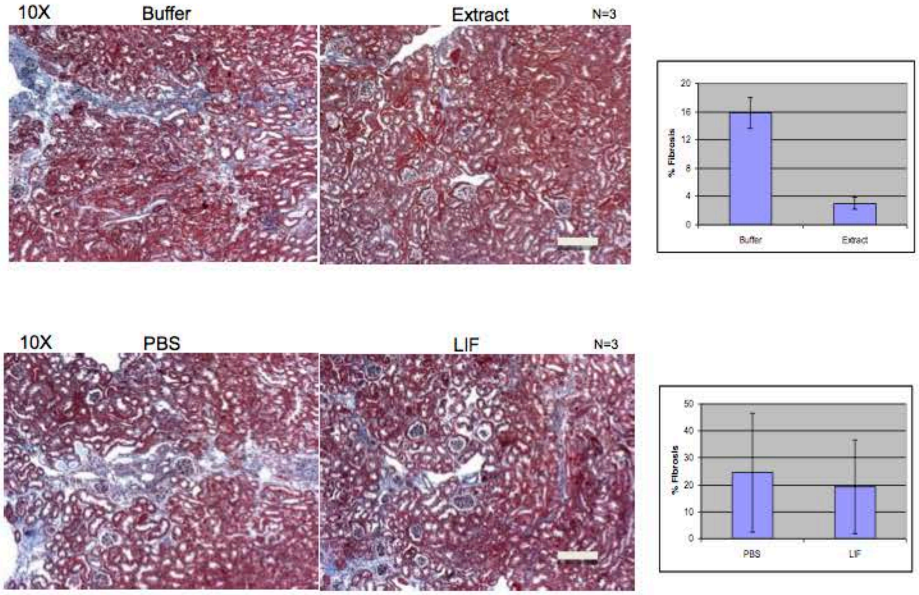

Suppl. Figure 5. Comparative analysis of Masson's trichrome staining of kidneys of mice with chronic folic acid-induced nephropathy treated with the EPC extract (vs buffer alone), upper panel, or with LIF, lower panel, all delivered by subcapsular injection of 10 ul/kidney. Summary of the extent of fibrosis is provided in the right-hand panels. Note that the injection of LIF did not result in detectable amelioration of fibrosis. Scale bar = 400 um.
